# Why a large percentage of Tunisian women aged 40 years and more has a reduced forced vital capacity? The implication of parity

**DOI:** 10.1186/s12890-022-02218-1

**Published:** 2022-11-11

**Authors:** Helmi Ben Saad

**Affiliations:** Laboratory of Physiology, Faculty of Medicine of Sousse, Farhat HACHED Hospital, Sousse, Research Laboratory Heart Failure, LR12SP09, University of Sousse, Street Mohamed KAROUI, 4000 Sousse, Tunisia

**Keywords:** Childbearing, Parity, FVC, Spirometry, Low-income country

## Abstract

The investigation of the link between reduced forced vital capacity (FVC) and risk factors and health variables in women aged ≥ 40 years is encouraged since a reduced FVC was related to all-cause mortality. The high frequency of women with a reduced FVC, observed in some studies, could be related to the impacts of parity on lung. In the literature, the association between parity and health consequences is discussed in terms of “selection pressure”, and the trade-off between longevity and fertility described by scientists is termed the “longevity determination” or “biological warranty period”. The respiratory system could be influenced by parity. Above all, it is the respiratory system, who endures the repercussions of the numerous physio-pathological experiences of the woman life. The probable effects of parity on lung function data, including FVC, make parity a key predictor to be stressed and evaluated. Parity is a promising original direction for physiological and pathophysiological research, particularly for low- and lower-middle- income countries. Thus, upcoming epidemiological and clinical studies of lung function data in women would need to include information about their parity status.

Dear editor,

I read with great interest the paper entitled “Reduced forced vital capacity is independently associated with, aging, height and a poor socioeconomic status: a report from the Tunisian population-based BOLD study” [1]. The investigation of the link between reduced forced vital capacity (FVC) and risk factors and health variables in an urban population of subjects aged ≥40 years and living in a low-income country, such as Tunisia, is encouraged since a reduced FVC was related to all-cause mortality [2]. In their paper, Hsan et al. [1] were surprised to note that 89.6% of women who had never smoked had a reduced FVC. They reported the following sentence “surprisingly, a large percentage of subjects with reduced FVC were never smokers (60.8%). This appears clearly especially in women with a reduced FVC since 89.6% of them had never smoked” [1]. The high frequency of women with a reduced FVC could be related to the impacts of parity (*ie*; the number of offspring a woman has borne) on lung [3]. On the one hand, contrary to high-income countries such as North American/European ones, parity is a particular topic in low- and lower-middle-income countries such as African ones [4]. For example, during 2015, while the mean of parity was 1.616 in European, it was 4.589 in Africa [4]. On the other hand, in the literature, the association between parity and health consequences is discussed in terms of “selection pressure” [5]. The respiratory system could be influenced by parity [3]. Above all, it is the respiratory system who endure the repercussions of the numerous physio-pathological experiences of the woman life [3]. It is surprising that Hsan et al. [1] “omitted” this crucial point (*ie*; the association between parity and FVC), especially since some of studies on this topic come from the same region (*ie*; Sousse, Tunisia) [3, 6–10]. The probable effects of parity on lung function data (LFD), including FVC, make parity a key predictor to be stressed and evaluated [3]. A 2021 Tunisian systematic review has reviewed the studies (*n* = 10) assessing the effects of parity on LFD of healthy and unhealthy women [3]. The authors of the retained 10 studies [6–15] in the aforementioned systematic review [3] have expressed parity as numerical and/or dichotomous data. For the dichotomous expression mode, parity was presented in terms of two groups or different classes, and multiple parity thresholds were applied to classify women into groups or classes [3].

The five studies including healthy women [6–9, 11] reported contradictory results regarding the effects of multiparity on LFD (Table 1). First, some studies reported no effect of multiparity on some LFD (*eg*; similar LFD between the groups and/or classes of women divided according their parity) [6–9]. Second, certain studies recorded positive effects of multiparity on many LFD (*ie*; multiparous women have larger forced expiratory volume in 1 s (FEV_1_), FVC, slow vital capacity, and inspiratory capacity) [9, 11]. Third, many studies noted that multiparity had negative effects on plenty LFD (*eg*; multiparous had higher static lung volumes; lower flows; tendencies to an obstructive impairment and/or lung-hyperinflation) [6, 7, 9]. Fourth, one study highlighted an acceleration of the lung aging process in multiparous women [10].

**Table 1:** Studies including healthy women and evaluating the effects of parity on lung function data

***1st author***	Harik-Khan [11]	Ben Saad [6]	Ben Saad [7]	Ben Saad [8]	Ketfi [9]
***Parity groups or classes***	**2 groups** G_1_:0:35^**a**^ G_2_:≥ 1: 65^**a**^ **4** ***classes*** C_1_:0: 48.70^**a**^ C_2_:1: 15.3^**a**^ C_3_:2: 19.7^**a**^ C_4_: ≥3:16.2^**a**^	**2 groups** G_1_:≤ 4: 22^**a**^ G_2_:> 4: 78^**a**^	**2 groups** G_1_:≤3: 49.1^**a**^ G_2_:≥4: 50.9^**a**^ **3** ***classes*** C_1_:< 2: 12.0^**a**^ C_2_:3–4: 58.3^**a**^ C_3_:> 4: 29.7^**a**^	**2 groups** G_1_:≤5: 50^**a**^ G_2_:≥6: 50^**a**^	**2 groups** G_1_:1–6: 51.5^**a**^ G_2_:7–14: 48.5^**a**^ **3** ***classes*** C_1_:1–4:22.7^**a**^ C_2_:5–8:42.4^**a**^ C_3_:9–14:34.8^**a**^
***Comparisons***	**G**_**2**_ **vs. G**_**1**_ .Higher FEV_1_ (%) **C**_**4**_ **or C**_**3**_ **or C**_**2**_ **vs. C**_**1**_ .Higher FEV_1_	**G**_**2**_ **vs. G**_**1**_ .Similar FVC .Lower FEV_1_, FEV_1_/FVC, MMEF, PEF	**G**_**2**_ **vs. G**_**1**_ .Similar values in women aged 45–49 or 50–54 or 55–59 years .Penchant to a bronchial obstruction (decrease of FEV_1_/SVC and FEV_1_/FVC) **C**_**2**_ **and C**_**3**_ **vs. C**_**1**_ .Penchant to a bronchial obstruction (decrease of FEV_1_/FVC and Gawtot) without an associated restriction	**G**_**2**_ **vs. G**_**1**_ .Similar spirometric data (absolute values)	**G**_**2**_ **vs. G**_**1**_ .Similar values of FVC, FEV_1_, MMEF, FEF_x%_, PEF, sRaw, FEV_1_/FVC; FEV_1_/SVC, RV/TLC .Higher TGV, RV, TLC **C**_**1**_ **vs. C**_**2**_ **and C**_**3**_ .Similar values of MMEF, FEF_x%_, PEF, TGV, ERV, IRV, FEV_1_/FVC; FEV_1_/SVC, RV/TLC **C**_**2**_ **vs. C**_**1**_ .Higher FEV_1_, FVC, SVC, TLC, IC, sRaw **C**_**3**_ **vs. C**_**1**_ .Higher TLC and RV

*C*_*x*_ Class number x, *ERV* Expiratory-reserve-volume, *G*_*x*_ Group number x, *FEF*_*X%*_ Forced-expiratory-flow when X% of FVC has been exhaled, *FEV*_*1*_ Forced-expiratory-volume in one second, *FVC* Forced-vital-capacity, *Gawtot* total-airway-conductance, *IC* Inspiratory capacity, *IRV* Inspiratory-reserve-volume, *MMEF* Maximal mid-expiratory flow, *PEF* Peak expiratory flow, *RV* Residual volume, *sRaw* Specific airway resistance, *SVC* Slow-vital-capacity, *TGV* Thoracic-gas-volume, *TLC* total-lung-capacity

^a^Data were percentage

The four studies [12–15] examining the effects of parity on the LFD of unhealthy women [*ie*; chronic obstructive pulmonary disease (COPD), defect in protease-inhibitor (Pi), certains chronic conditions (COPD, asthma, endometriosis, ovarian diseases)] or a mixed population of healthy/unhealthy women also reported opposing outcomes (Table 2). On the one hand, certain studies stated that parity has no effect on FEV_1_ decline or the natural history of COPD with comparable *i)* initial mean FEV_1_, *ii)* rate of FEV_1_ decline; and *iii)* COPD incidence between the different groups of parity [12], or that there is no association between parity and FEV_1_ or FEV_1_/FVC in never-smokers or in those with low smoking exposure [15]. On the other hand, three studies identified negative effects of parity on certain LFD (*eg*; multiparous women have a tendency to an obstructive impairment; lower flows; lower post-bronchodilator FEV_1_, and increased Pi levels) [13–15].

**Table 2:** Studies including unhealthy women or mixed population of unhealthy and healthy women and evaluating the efects of parity on the spirometric data

***1st author***	Krzyzanowski [12]	Horne [13]	Tang [14]	Moll [15]		
***Parity groups or classes***	**5 groups** G_1_:0:14.7^**a**^ G_2_:1:21.4^**a**^ G_3_:2:40^**a**^ G_4_:3:16.5^**a**^ G_5_:≥ 4:7.4^**a**^ **COPD: 5 classes** C_1_:0:19.6^**a**^ C_2_:1:11,8^**a**^ C_3_:2:45.1^**a**^ C_4_:3:9.8^**a**^ C_5_:> 3:13.7^**a**^	**Parity** .2.4^**b**^: non-S Pi MZ .2.6^**b**^: non-S Pi M .2.6^**b**^: ever-S Pi MZ .2.8^**b**^: ever-S Pi M .2.5^**b**^: Pi MZ .2.7^**b**^: Pi M	**2 groups** G_1_:0:15.0^**a**^ G_2_:≥1:85^**a**^ **5 classes** C_1_:0:15.0^**a**^ C_2_:1:12.2^**a**^ C_3_:2:34.4^**a**^ C_4_:3:25.3^**a**^ C_5_:> 3:13.2^**a**^	**2 groups** G_1_:0:(control:23.5^**a**^, PRISm:11.4^**a**^, COPD:17.4^**a**^) G_2_:≥1:(control:76.5^**a**^, PRISm:78.6^**a**^, COPD:82.6^**a**^) **3 classes** C_1_:≤1:(control:23.5^**a**^, PRISm:21.4^**a**^, COPD:17.4^**a**^) C_2_:2:(control:34.4^**a**^, PRISm:27.3^**a**^, COPD:33.4^**a**^) C_3_:≥3:(control:42.1^**a**^, PRISm:51.4^**a**^, COPD:49.2^**a**^)	**COPDGene**	**NHANES 2011–2012 dataset**
					**2 groups** G_1_:0:(never-S:12.1^**a**^, LSE:11.3^**a**^) G_2_:≥1:(never-S:87.9^**a**^, LSE:88.7^**a**^)	**3 classes** C_1_:≤1:(never-S:12.1^**a**^, LSE:11.3^**a**^) C_2_:2: (never-S:38.9^**a**^, LSE:39.2^**a**^) C_3_:≥3: (never-S:48.9^**a**^, LSE:49.5^**a**^)
***Results***	**Comparison between the 5 groups** .Similar initial mean FEV_1_ .Similar rate of FEV_1_ decline (mL/Y) **Regression analysis** .Faster FEV_1_ decline in women reporting a greater parity (regression coefficient = 1.6) **COPD incidence** .Similar incidence between the 5 classes	.Parity/Healthy women: not associated with residual pulmonary function values .Interaction term for Pi MZ and parity (***parity x MZ***): associated with the residuals pulmonary function values: FEV_1_/FVC, MMEF, FEF_50%_, FEF_25%_ values increase with the increasing parity in the Pi MZ group not in the Pi M group .Interaction term for parity and history of smoking (***smoking x parity***): associated with FEF_25%_: it increases rapidly with increasing parity in Pi MZ group with a history of smoking than in Pi MZ group with no history of smoking .Pi MZ women with parity = 0 or 1 have lower flow rates than expected or that observed in healthy women .Pi MZ women with parity ≥3: higher flow rates than expected	**G**_**1**_ **vs. G**_**2**_ .Lower FEV_1_, FVC .Greater FEV_1_/FVC **C**_**1**_ **vs. C**_**2**_ .Lower FVC, similar FEV_1_ .Greater FEV_1_/FVC **C**_**1**_ **vs. C**_**2**_ **(or C**_**3**_**, C**_**4**_**, C**_**5**_**)** .Lower FEV_1_, FVC .Greater FEV_1_/FVC	**Overall analysis** .C_2_ and C_3_: association with a lower FEV_1_ (%) .G_2_: association with a lower FEV_1_ (%) **Stratified analyses** .G_2_, C_2_ and C_3_: association with a lower FEV_1_ (%) for control and PRISm or mild obstruction but not across the more severe GOLD grades. **Interaction analyses** .Interaction of multiparity with age on FEV_1_ (%) .No interaction of multiparity with smoking on FEV_1_ (%)	.Multiparity: no association with FEV_1_ (%) or FEV_1_/FVC in never-S or LSE groups	

*COPD* Chronic obstructive pulmonary disease, *COPDGene* COPD Genetic Epidemiology, *C*_*x*_ Class number x, *ever-S* Ever-smokers, *FEF*_*x%*_ Forced-expiratory-flow when x% of FVC has been exhaled, *FEV*_*1*_ Forced-expiratory-volume in one second, *FVC* Forced-vital-capacity. *GOLD* Global obstructive lung disease, *G*_*x*_ Group number x, *LSE* Low smoking exposure, *MMEF* Maximal-mid-expiratory-flow, *never-S* Never-smokers, *NHANES* National Health and Nutrition Examination Survey, *non-S* Non-smokers, *Pi* Protease-inhibitor, *PRISm* Impaired spirometry, *ERV* Expiratory-reserve-volume, *G*_*x*_ Group number x

Data were: ^a^Percentage. ^b^Mean

To conclude, parity is a promising original direction for physiological and pathophysiological research, particularly for low- and lower-middle- income countries [3]. In the litterature, the trade-off between longevity and fertility described by scientists is termed the “longevity determination” or “biological warranty period” [16]. A negative phenotypic correlation is recognized between parity and lifespan, with multiparous women being more probably to have shorter lifespans [17]. Moreover, multiparity is linked with frequent poor outcomes, such as faster markers of aging (*eg*, telomere length), oxidative stress, coronary heart conditions, stroke, heart-failure, arterial-hypertension, type 2 mellitus-diabetes, renal cancer, and bad health related quality-of-life [18, 19]. In the study of Hsan et al. [1], multiparity could partly explicate the reported high frequency of women with a reduced FVC. Thus, upcoming epidemiological and clinical studies of LFD in women would need to include information about their parity status.

## Data Availability

Not applicable.

## Abbreviations

COPD: Chronic obstructive pulmonary disease
FEV_1_: Forced expiratory volume in one second
FVC: Forced vital capacity
LFD: Lung function data
Pi: Protease-inhibitor
